# Genotyping of *RB1* status identifies two distinct subtypes in *EGFR*‐mutant lung cancers with SCLC transformation

**DOI:** 10.1002/ctm2.1683

**Published:** 2024-05-12

**Authors:** Jie Huang, Shi‐Ling Zhang, Chao Zhang, Weiye Huang, Zhenhua Zhang, Yu‐Qing Chen, Jun‐Wei Su, Hong‐Hong Yan, Hua‐Jun Chen, Jin‐Ji Yang, Junjian Wang, Yi‐Long Wu

**Affiliations:** ^1^ Guangdong Lung Cancer Institute, Guangdong Provincial Key Laboratory of Translational Medicine in Lung Cancer Guangdong Provincial People's Hospital, Guangdong Academy of Medical Sciences, Southern Medical University Guangzhou China; ^2^ Department of Medical Oncology Affiliated Cancer Hospital and Institute of Guangzhou Medical University Guangzhou China; ^3^ Department of Pathology Guangdong Provincial People's Hospital, Guangdong Academy of Medical Sciences, Southern Medical University Guangzhou China; ^4^ School of Pharmaceutical Sciences Sun Yat‐sen University Guangzhou China; ^5^ National‐Local Joint Engineering Laboratory of Druggability and New Drugs Evaluation Guangdong Provincial Key Laboratory of New Drug Design and Evaluation, Sun Yat‐sen University Guangzhou China

Dear Editor,

In this study, we investigate the significant yet underexplored clinical and molecular heterogeneity in epidermal growth factor receptor (*EGFR)‐*mutant lung cancers transformed into small cell lung cancer (SCLC). Our work addresses a crucial unmet need in understanding the molecular underpinnings of this transformation and introduces a novel predictive model for assessing the transformation hazards in high‐risk patients.

Up to 14% of *EGFR*‐mutant non‐SCLC (NSCLC) patients experience SCLC transformation (T‐SCLC) under the selective pressure of EGFR tyrosine kinase inhibitors (TKIs),^1^ and the significant heterogeneity within this population is increasingly recognised, including response to EGFR‐TKIs, time to transformation, survival and genetic profile.^2^, ^3^, ^4^ However, this patient population is generally treated as a homogeneous entity, yet their post‐transformation prognosis is often worse than that of de novo SCLC.^4^, ^5^ Hence, it is rational to define *EGFR*‐mutant lung cancer with T‐SCLC into distinct subtypes, potentially uncovering new therapeutic targets and vulnerabilities.

We conducted a retrospective analysis of 1436 *EGFR*‐mutant NSCLC patients at the Guangdong Lung Cancer Institute between January 2017 and December 2021. This cohort included 63 patients who underwent T‐SCLC and 97 patients who harboured concomitant *RB1* and *TP53* alterations along with *EGFR* mutation (Supporting Information Figure S1). Furthermore, T‐SCLC patients were stratified into two subgroups based on pre‐transformation *RB1* status: the *EGFR*‐mutant/*RB1*‐wild subgroup (*n* = 21) and the *EGFR*/*RB1*‐mutant subgroup (*n* = 34). Additionally, *EGFR/RB1/TP53*‐mutant patients were categorised into the transformed subgroup (*n* = 34) and the untransformed subgroup (*n* = 27). Notably, the transformed subgroup completely overlapped with the *EGFR*/*RB1*‐mutant subgroup, as all the patients in the *EGFR*/*RB1*‐mutant subgroup harboured *TP53* mutations simultaneously (Supporting Information: Methods).

Baseline demographics of these subgroups are shown in Table 1. It is well known that *RB1* inactivation favours T‐SCLC in *EGFR*‐mutant tumours.^6^ Unexpectedly, the *EGFR/RB1*‐mutant subgroup demonstrated a significantly longer median time to transformation than the *EGFR*‐mutant/*RB1*‐wild subgroup (Figure 1A). Patients with the T790M mutation or the *SMAD4* mutation also showed a significantly prolonged time to transformation (Figure 1B,C; Supporting Information Table S1). However, multivariate analysis identified *RB1* and SMAD4, not T790M status, as independent prognostic factors for transformation (Figure 1D; Supporting Information Table S2). Of note, the number of *SMAD4*‐mutant patients was limited (*n* = 2). This suggests that some other mechanisms rather than *RB1* mutation might drive T‐SCLC in the *EGFR*‐mutant/*RB1*‐wild subgroup. Post‐transformation survival and overall survival did not differ between the subgroups (Supporting Information Figure S2A–D).

**TABLE 1:** Comparison of characteristics of 82 enrolled patients.

Characteristics	*EGFR*‐mutant/*RB1*‐wild subgroup (*N* = 21)	*EGFR/RB1*‐mutant subgroup/ Transformed subgroup (*N* = 34)	Untransformed subgroup (*N* = 27)	*P*1	*P*2
Gender				.269	.375
Male	8	19	12		
Female	13	15	15		
Age	53 (32–72)	55 (24–72)	58 (35–85)	.765	**.023**
Smoking				.127	.559
Yes	2	9	9		
No	19	25	18		
*EGFR* mutation type				.795	**.016**
19DEL	15	26	12		
L858R	5	6	15		
G719	0	1	0		
L858R+T790M	1	1	0		
T790M				**.004**	**.010**
Yes	8	26	12		
No	13	8	15		
Staging				.485	.818
I	0	1	1		
II	0	1	2		
III	5	4	2		
IV	16	28	22		
First line TKI				.240	**.036**
1st generation	12	26	20		
2nd generation	5	3	7		
3rd generation	4	5	0		
Lines of pre‐T TKI				.052	
1	11	9			
≥2	10	25			
3rd generation TKI before T				.341	
No	6	6			
Yes	15	28			

Abbreviations: P1, the *EGFR*‐mutant/*RB1*‐wild subgroup versus the *EGFR*/*RB1*‐mutant subgroup; P2, the transformed subgroup versus the untransformed subgroup; TKI, tyrosine kinase inhibitor; T, transformation.

**FIGURE 1:**
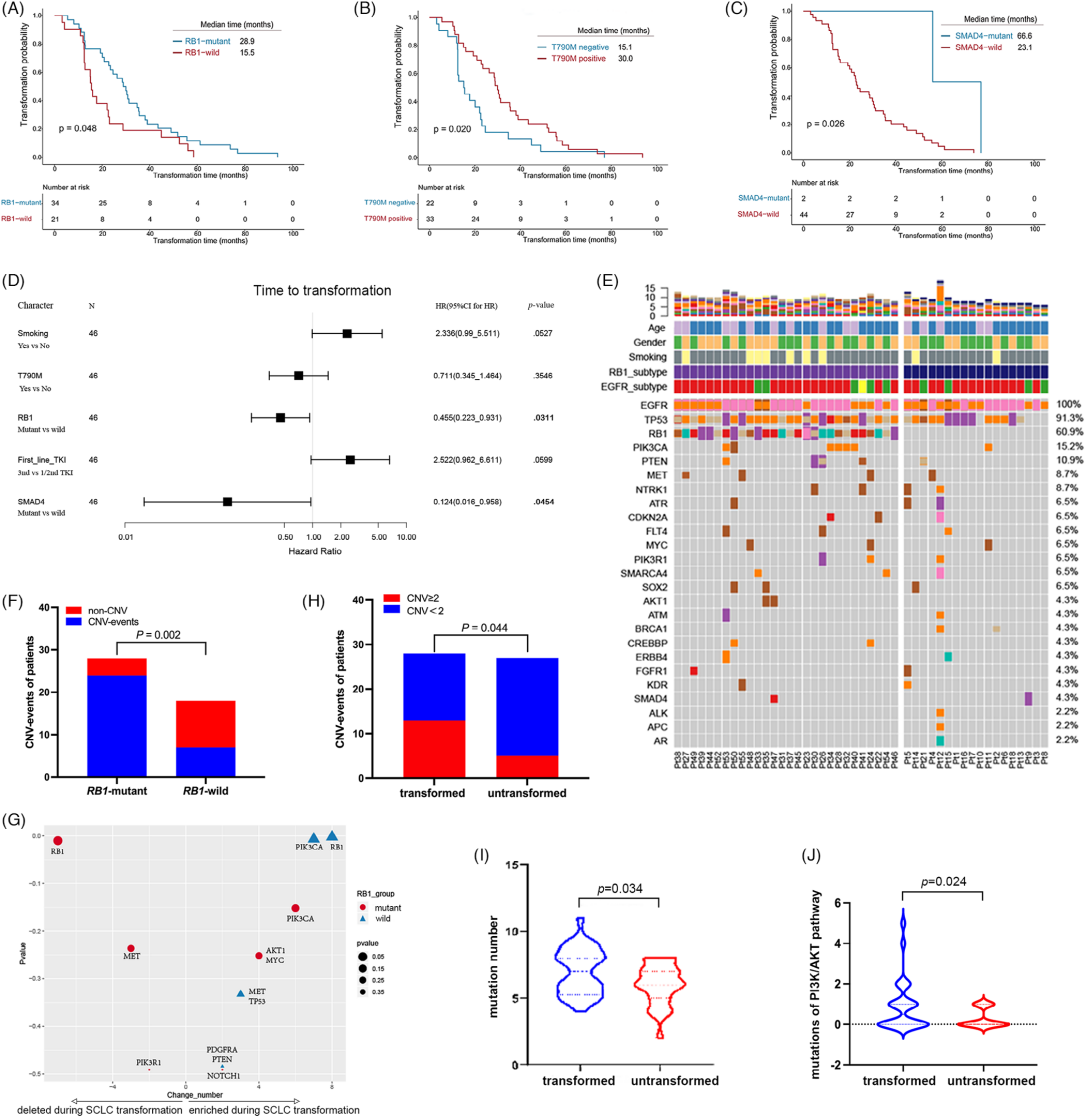
Clinical and genomic comparison between subgroups of epidermal growth factor receptor (*EGFR*)‐mutant lung cancer patients with small cell lung cancer (SCLC) transformation and *EGFR/RB1/TP53*‐mutant non‐SCLC (NSCLC) patients. (A) Time to transformation of the *EGFR/RB1*‐mutant subgroup and the *EGFR*‐mutant/*RB1*‐wild subgroup. (B) Time to transformation of T790M‐positive subgroup versus negative subgroup. (C) Time to transformation of *SMAD4*‐wild subgroup versus mutant subgroup. (D) Forest plot for transformation‐free survival. (E) Baseline genomic landscape of *EGFR*‐mutant lung cancer with SCLC transformation (T‐SCLC). (F) Comparison of the frequency of copy number variation (CNV) between the *EGFR/RB1*‐mutant subgroup and the *EGFR*‐mutant/*RB1*‐wild subgroup. (G) Enrichment analysis of genomic alterations during transformation in the *EGFR/RB1*‐mutant subgroup and the *EGFR*‐mutant/*RB1*‐wild subgroup. (H) The frequency of CNV (≥2/ < 2) in transformed and untransformed subgroups of *EGFR/RB1/TP53*‐mutant NSCLC patients. (I) Overall mutations in transformed and untransformed subgroups. (J) Mutations of phosphatidylinositol 3‐kinase (PI3K/AKT) pathway in transformed and untransformed subgroups.

Genomic analysis showed that despite no significant pre‐transformation mutational differences between the subgroups, the *EGFR/RB1*‐mutant subgroup, similar to de novo SCLC, had a higher frequency of copy number variations (CNVs) than the *EGFR*‐mutant/*RB1*‐wild subgroup (Figure 1E,F). In the analysis of matched pre‐ and post‐transformation samples, *PIK3CA* alterations were prevalent post‐transformation across both subgroups, while *AKT1* and *MYC* mutations were specifically enriched in the *EGFR/RB1*‐mutant subgroup (Figures 1G and S2E). Additionally, in *EGFR/RB1/TP53*‐mutant patients, those in the transformed subgroup exhibited a higher mutation load, particularly in the phosphatidylinositol 3‐kinase (PI3K)/AKT pathway, and demonstrated a more frequent CNVs≥ 2 than the untransformed subgroup (Figures 1H–J and S2F).

Further transcriptomic analysis revealed that pre‐transformation genes and pathways associated with neuroepithelial cell differentiation and ion regulation were up‐regulated in the *EGFR/RB1*‐mutant subgroup, while immune‐related genes and pathways were significantly suppressed, compared to the *EGFR*‐mutant/*RB1*‐wild subgroup, indicating a closer association with SCLC (Figure 2A‐C). Further comparing paired pre‐ and post‐transformation *EGFR*‐mutant/*RB1*‐wild samples, we observed a significant downregulation of tumour necrosis factor (TNF) signalling, hypoxia‐inducible factor 1 (HIF‐1) signalling and T‐cell differentiation pathways, whereas neuroactive ligand–receptor interaction and stem cell regulation pathways were more activated post‐transformation. Notably, PI3K/AKT signalling was inhibited, and no significant change was observed in the EGFR signalling pathway upon T‐SCLC, suggesting a low dependence on EGFR and PI3K/AKT pathways in the *EGFR*‐mutant/*RB1*‐wild subgroup (Figure 2D; Supporting Information Table S3).

**FIGURE 2:**
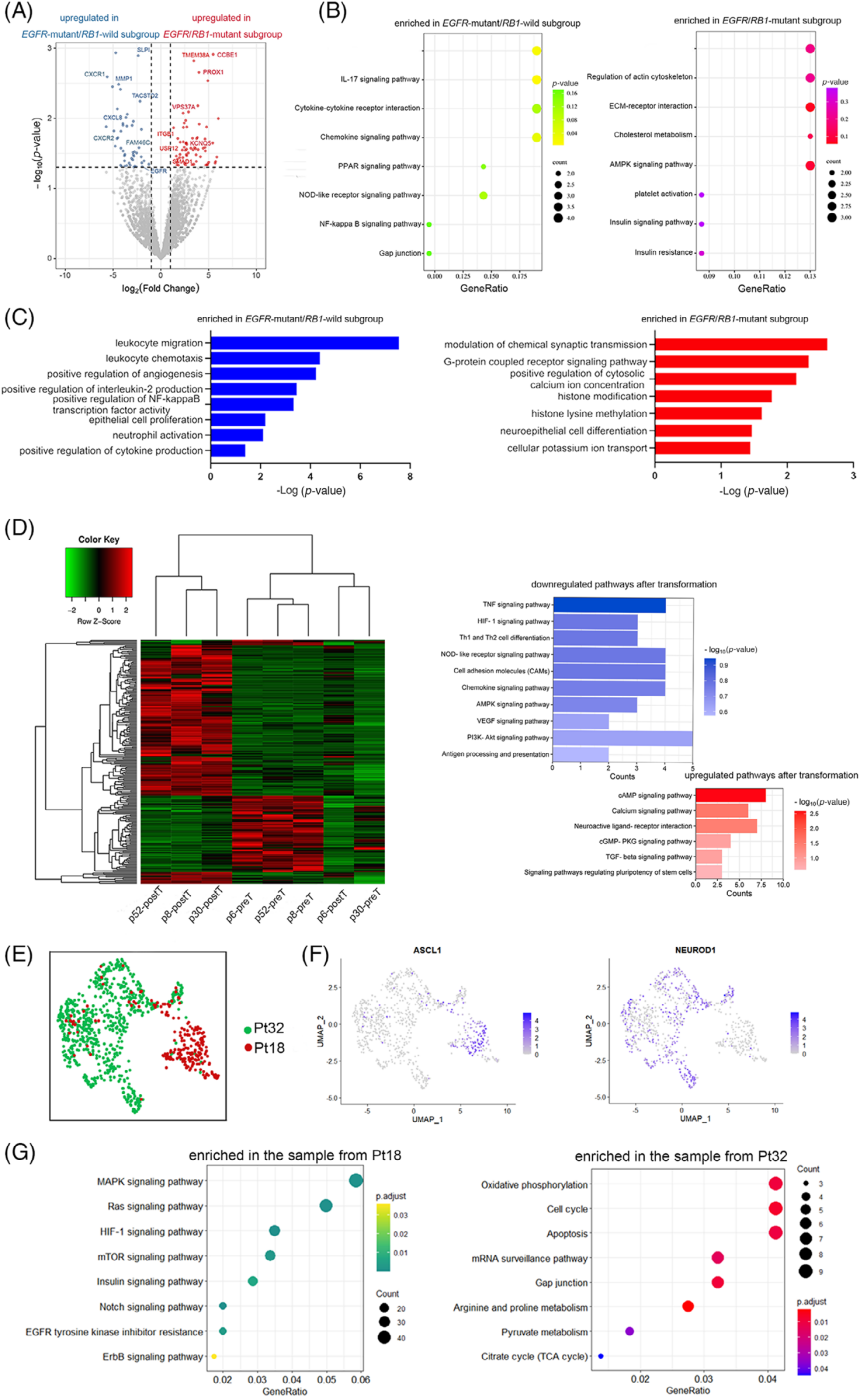
Transcriptomic analysis and the single‐cell RNA sequencing analysis of *EGFR*‐mutant lung cancer patients with T‐SCLC. (A) A volcano plot of differentially expressed genes (DEGs) between *EGFR*‐mutant/*RB1*‐wild subgroup and *EGFR/RB1*‐mutant subgroup. (B) Pathway enrichment analyses on the DEGs between the *EGFR*‐mutant/*RB1*‐wild subgroup and the *EGFR/RB1*‐mutant subgroup. (C) Gene Ontology analysis of differentially enriched biological processes between the *EGFR*‐mutant/*RB1*‐wild subgroup and the *EGFR/RB1*‐mutant subgroup. (D) Unsupervised clustering heatmap of DEGs and differentially regulated pathways after transformation in *EGFR*‐mutant/*RB1*‐wild lung cancers. (E) Uniform manifold approximation and projection (UMAP) plots of malignant cells colour‐coded by patients. (F) Feature plots of ASCL1 and NEUROD1 RNA expression levels across all the clusters. (G) GSEA of differentially enriched pathways between the *EGFR*‐mutant/*RB1*‐wild sample and the *EGFR/RB1*‐mutant sample. Pt32, patient 32 from the *EGFR/RB1*‐mutant subgroup; Pt18, patient 18 from the *EGFR*‐mutant/*RB1*‐wild subgroup.

Next, we used single‐cell RNA sequencing to analyze two post‐transformation samples from the *EGFR*‐mutant/*RB1*‐wild subgroup (Pt18) and the *EGFR/RB1*‐mutant subgroup (Pt32; Figure 2E). A total of 737 malignant cells were identified and classified into five distinct clusters (Supporting Information Figure S3). According to the expression of four classical transcriptional regulators, the cancer cells from Pt18 predominantly aligned with the *ASCL1* subtype, characterised by Notch and HIF‐1 signalling pathways, while the sample from Pt32 corresponded to the *NEUROD1* subtype, marked by significant upregulation of oxidative phosphorylation and cell cycle pathways (Figure 2F,G).

Although NSCLC patients harbouring triple *EGFR/RB1/TP5*3‐mutations have an elevated risk of transformation to SCLC, only 18% of them actually undergo transformation.^7^ Through comparative analysis of clinical profiles and molecular signatures between the transformed and the untransformed subgroups, we identified independent risk factors for transformation, including age, *EGFR* mutation type, CNV events and mutations of the PI3K/AKT pathway (Supporting Information Table S4). Based on these, a nomogram model was developed to predict the SCLC‐transformation risk (Figure 3A–D). The model's robustness was further validated in an external cohort, including three transformed and five untransformed cases, demonstrating its certain reliability (Figure 3E). Currently, an active clinical trial is evaluating the efficacy of osimertinib and chemotherapy in delaying T‐SCLC in *EGFR/RB1/TP5*3‐mutant patients (NCT03567642). This model might help to identify the beneficiaries of combination treatment.

**FIGURE 3:**
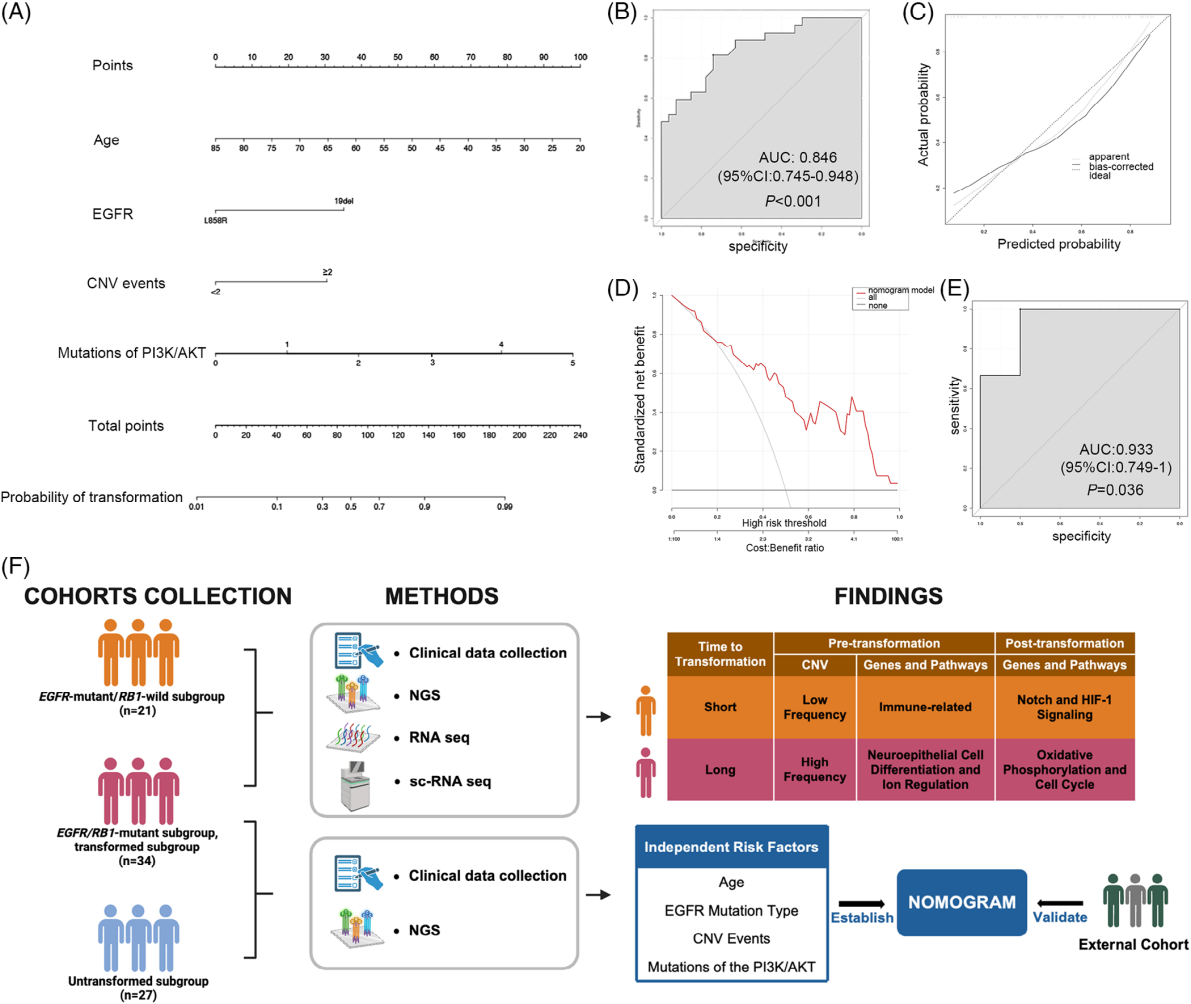
A prediction nomogram model in *EGFR/RB1/TP53* lung cancer patients. (A) Nomogram to predict the risk of transformation. (B) Receiver operating characteristic (ROC) curves of the nomogram in the training cohort. (C) Calibration plots of the nomogram. (D) Decision curve analysis (DCA) for the nomogram. (E) ROC curves of the nomogram in the external validation cohort. (F) The graphic abstract of the study, including cohorts collection, methods and findings.

To our knowledge, this is the first study to define subtypes of T‐SCLC, offering important perspectives on the disparate pathogenesis and potential therapeutic targets. The *EGFR*‐mutant/*RB1*‐wild subgroup exhibited a more active immune microenvironment, which became markedly ‘colder’ after transformation, indicating that immunotherapy might be an appropriate therapy. Our previous retrospective study revealed that immunochemotherapy, with or without bevacizumab, might be a promising approach for treating T‐SCLC.^8^ In contrast, the *EGFR/RB1*‐mutant subgroup exhibited a marked increase in PI3K/AKT pathway mutations post‐transformation, suggesting that PI3K/AKT pathway activation might be essential for transformation, consistent with previous findings.^9^, ^10^ Consequently, this subgroup might be sensitive to AKT inhibitors, such as samotolisib.

In conclusion, our study comprehensively characterises T‐SCLC patients, introducing a novel classification based on *RB1* status (Figure 3F). Our results deepen the understanding of molecular biology underlying T‐SCLC and pave the way for personalised treatment strategies. In addition, our predictive nomogram model significantly improves the accuracy of assessing the SCLC‐transformation risk in *EGFR/RB1/TP53*‐mutant NSCLCs, highlighting the need for further validation.

## AUTHOR CONTRIBUTIONS

*Conception and design*: Junjian Wang, Yi‐Long Wu and Jie Huang. *Development of methodology*: Jie Huang, Chao Zhang and Shi‐Ling Zhang. *Acquisition of data*: Weiye Huang, Zhenhua Zhang, Yu‐Qing Chen and Jun‐Wei Su. *Analysis and interpretation of data*: Jie Huang, Hong‐Hong Yan, Hua‐Jun Chen and Jin‐Ji Yang. *Writing, review and/or revision of the manuscript*: Junjian Wang, Yi‐Long Wu, Jie Huang, Chao Zhang and Shi‐Ling Zhang. *Administrative, technical or material support*: Hong‐Hong Yan, Hua‐Jun Chen and Jin‐Ji Yang. *Study supervision*: Junjian Wang and Yi‐Long Wu. All authors read and approved the final version of the manuscript. Jie Huang, Shi‐Ling Zhang and Chao Zhang have accessed and verified the data. Junjian Wang and Yi‐Long Wu were responsible for the decision to submit the manuscript.

## CONFLICT OF INTEREST STATEMENT

All the authors declare that they have no conflicts of interest.

## FUNDING INFORMATION

The High‐level Hospital Construction Project (Grant Number DFJH201917); Guangdong Association of Clinical Trials (GACT)/Chinese Thoracic Oncology Group (CTONG) and Guangdong Provincial Key Lab of Translational Medicine in Lung Cancer (Grant Number 2017B030314120); Guangdong Basic and Applied Basic Research Foundation (Grant Number 2019B151502016, 2022B1515130008)

## ETHICS STATEMENT

This study was approved by the research ethics committee of Guangdong Provincial People's Hospital, Guangdong Academy of Medical Sciences, and carried out under the World Medical Association Declaration of Helsinki.

## Supporting information

Supporting Information

Supporting Information

Supporting Information

## Data Availability

Sequencing data and code for this study can be obtained by contacting the corresponding author upon reasonable request.
