# Supplementary material for: Genotyping of RB1 status identifies two distinct subtypes in EGFR‐mutant lung cancers with SCLC transformation
Source: Clin Transl Med. 2024 May 12;14(5):e1683. doi: 10.1002/ctm2.1683 (PMC11089080; doi:10.1002/ctm2.1683)
Supplement: Supplementary file 1 — Supporting Information [file CTM2-14-e1683-s002.docx]

**Supplementary Figures**

**Supplementary Figure 1.** Schematic diagram of patient screening, patient grouping, clinical and molecular comparison, and the nomogram model construction.


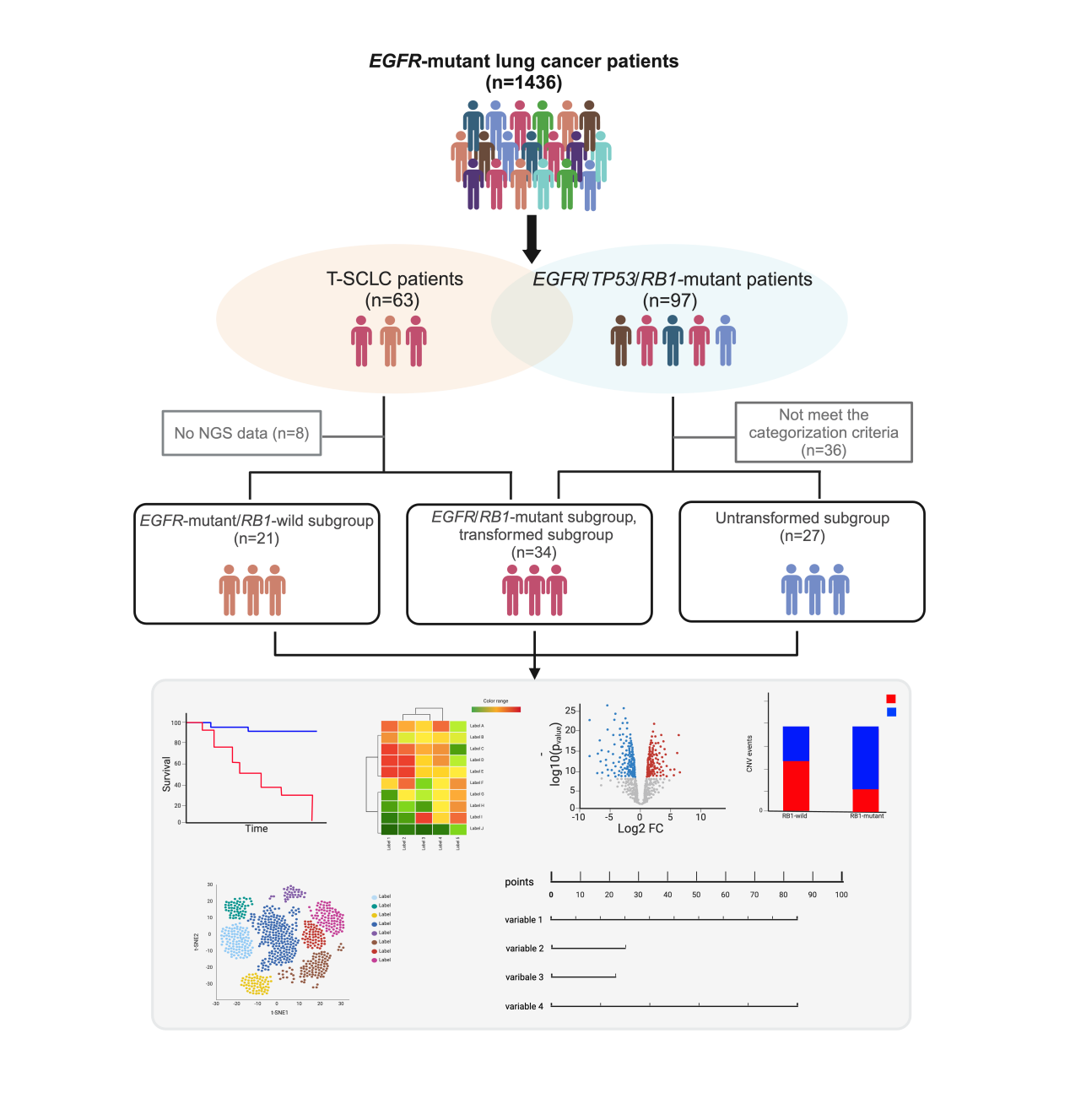


**Supplementary Figure 2.** Survival and genomic analyses of *EGFR*-mutant lung cancer patients with SCLC transformation and *EGFR/RB1/TP53*-mutant non-small cell lung cancer (NSCLC) patients.


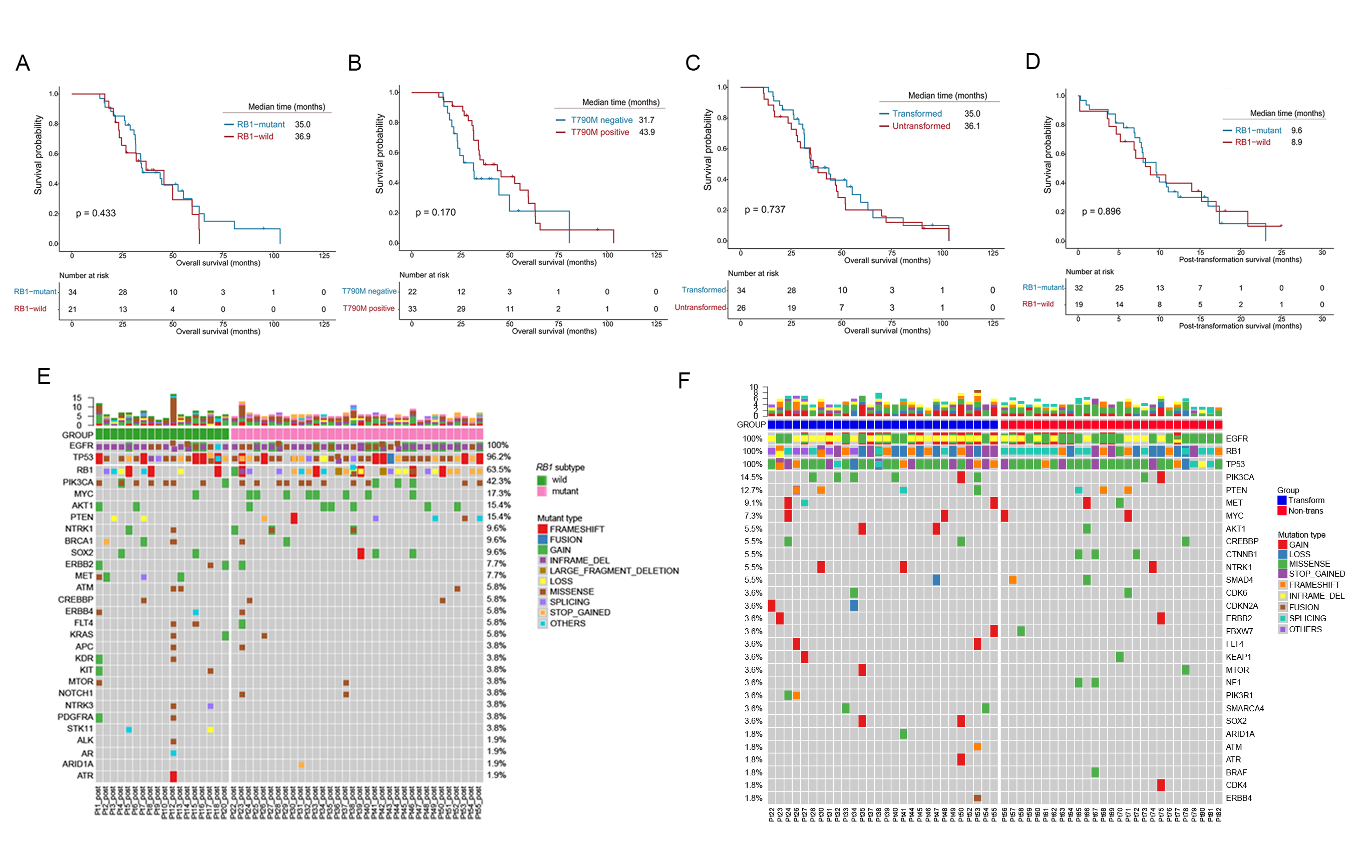


1. Overall survival (OS) of *EGFR*-mutant/*RB1*-wild subgroup versus *EGFR/RB1*-mutant subgroup.
2. OS of patients with or without T790M mutations.
3. OS of transformed subgroup versus untransformed subgroup of *EGFR/RB1/TP53*-mutant lung cancers.
4. Post-transformation survival of *EGFR*-mutant/*RB1*-wild subgroup versus *EGFR/RB1*-mutant subgroup.
5. Genomic landscape of *EGFR*-mutant lung cancer after transformation defined by next-generation sequencing (NGS).
6. Genomic landscape of transformed subgroup and untransformed subgroup in *EGFR/RB1/TP53*-mutant NSCLC patients defined by NGS.

**Supplementary Figure 3.** Tumor heterogeneity of post-transformation SCLC at single-cell resolution.


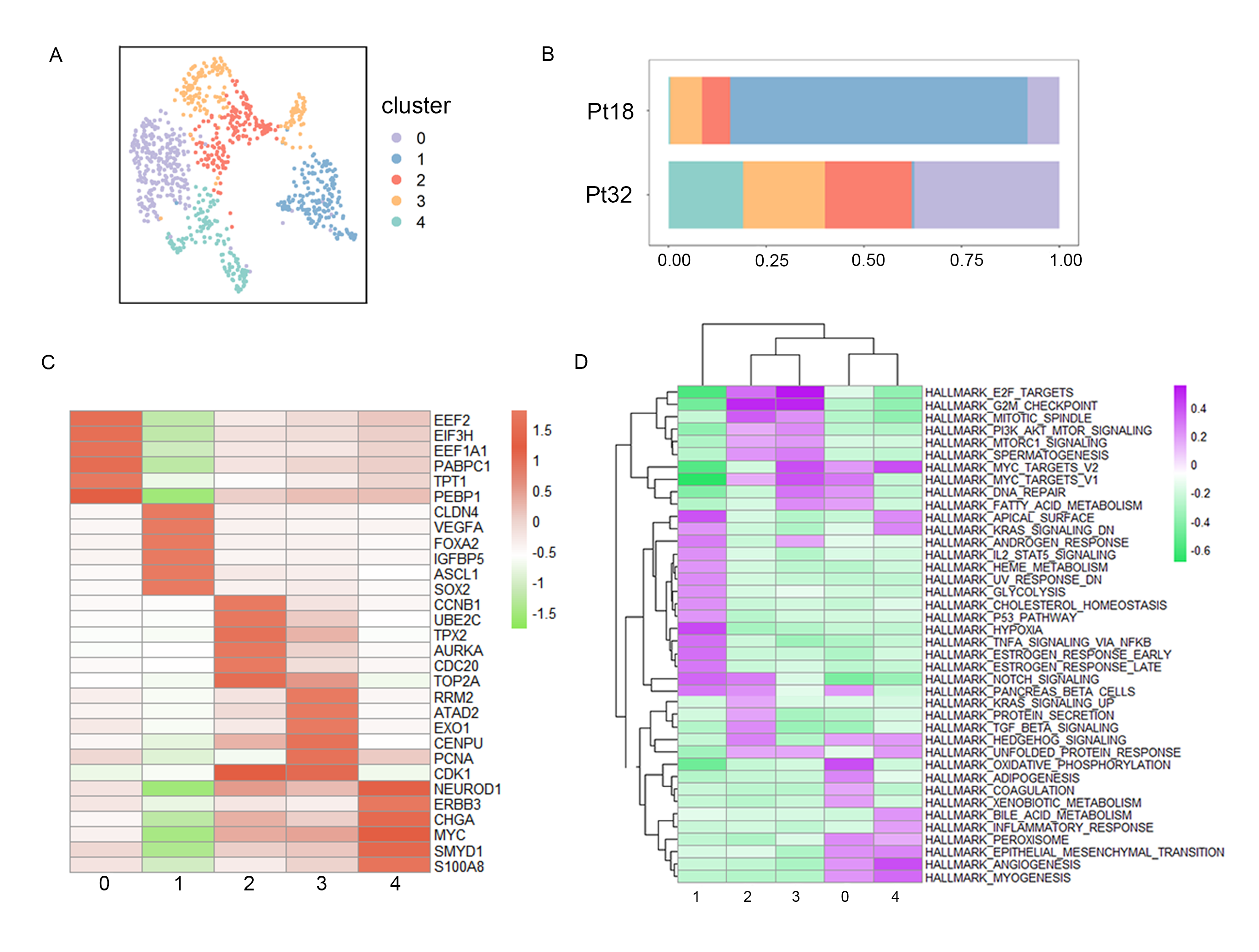


1. Uniform manifold approximation and projection (UMAP) plots of malignant cells color-coded by clusters.
2. Proportions of five clusters in individual samples.
3. Heatmap for the RNA expression levels of marker genes in each cluster.
4. Heatmap showing different pathways enriched in each cluster by gene set variation analysis (GSVA) analysis, colored by Z-score transformed mean GSVA scores.
