# Supplementary material for: Genotyping of RB1 status identifies two distinct subtypes in EGFR‐mutant lung cancers with SCLC transformation
Source: Clin Transl Med. 2024 May 12;14(5):e1683. doi: 10.1002/ctm2.1683 (PMC11089080; doi:10.1002/ctm2.1683)
Supplement: Supplementary file 3 — Supporting Information [file CTM2-14-e1683-s003.docx]

**Supplementary Methods**

**Patient selection and data collection**

A single-center retrospective analysis was conducted on *EGFR*-mutant NSCLC patients' records from January 2017 to December 2021 at the Guangdong Lung Cancer Institute. We utilized next-generation sequencing (NGS) or amplification refractory mutation system-polymerase chain reaction (ARMS-PCR) to identify *EGFR* mutations in all cases. Among them (n=1436), we selected individuals with either SCLC transformation (n=63, 4%) or concurrent *EGFR/RB1/TP53* mutations (n=97, 7%) for further analyses. SCLC transformation was diagnosed by two independent pathologists based on histology or cytology specimens with routine hematoxylin and eosin staining according to the 2015 WHO classification^1^. Immunohistochemistry (IHC) assays were required when necessary to confirm equivocal features or address differential diagnoses, especially in small biopsies with crush artifacts^2^. IHC markers and their characteristics for T-SCLC diagnosis are the same as that for SCLC. NGS was employed to detect alterations in *RB1* and *TP53*.

SCLC-transformed patients were stratified into two subgroups based on pre-transformation *RB1* status from NGS results: the *EGFR*-mutant/*RB1*-wild subgroup (n=21, 33%) and the *EGFR*/*RB1*-mutant subgroup (n=34, 54%). Eight patients were excluded from subgroup analysis due to the unavailability of NGS data. Ninety-seven *EGFR/RB1/TP53*-mutant patients were categorized based on whether they underwent SCLC transformation. The patients with tissue rebiopsy pathology confirming SCLC transformation were referred to as the transformed subgroup (n=34, 35%). Considering that the SCLC transformation is a relatively long process and the median time to transformation was reported to be 17.8 months^3^, the untransformed subgroup (n=27, 28%) was defined based on more strict criteria: 1) the tissue rebiopsy is required to be performed at least 18 months after initial diagnosis of NSCLC or within one month before death; 2) the pathology of tissue rebiopsy confirmed no SCLC transformation. We excluded 36 patients who did not meet these criteria.

NGS data were collected from 73 pre-transformation tumor tissue or plasma samples and 52 post-transformation tumor tissue or plasma samples. Clinical data collection from 82 enrolled patients encompassed demographic and clinical variables, including gender, age, smoking status, staging, *EGFR* mutation status, T790M status, treatment regimens (initial EGFR-TKI therapy and lines of pre-transformation EGFR-TKIs), and outcomes. Time to transformation was defined as the time from initial NSCLC diagnosis to histologically confirmed SCLC transformation.

This study was undertaken with the approval of the Institutional Review Board and the Research Ethics Committee of the Guangdong Provincial People’s Hospital, Guangdong Academy of Medical Sciences (Guangzhou, China).

**Sample collection**

Tumor tissues from 82 patients were collected by tissue biopsy and prepared into formalin-fixed paraffin-embedded (FFPE) samples. Genomic DNA and total RNA extraction from FFPE samples were performed for NGS and RNA-sequencing. Additionally, fresh tumor tissues were obtained from metastatic lymph node biopsy of two patients with SCLC transformation for single-cell RNA sequencing. Peripheral blood (10 mL) was collected from each patient in EDTA-coated tubes (BD biosciences). Plasma was extracted within 2 hours of blood collection and dispatched to the laboratory for NGS analysis within 48 hours.

**NGS and RNA-sequencing**

Comprehensive genomic profiling was performed with NGS in two laboratories: Burning Rock Biotech (Guangzhou, China) and Nanjing Geneseeq Technology Inc. (Nanjing, China). Genomic DNA from FFPE sections and whole blood control samples were extracted with QIAamp DNA FFPE Tissue kit and DNeasy Blood and tissue kit (Qiagen, USA), respectively. Circulating cell-free DNA (cfDNA) from plasma was extracted using the QIAamp Circulating Nucleic Acid kit (Qiagen). Sequencing libraries were prepared using the KAPA Hyper Prep Kit (KAPA Biosystems) according to the manufacturer's instructions for different sample types. Customized xGen lockdown probes panel (Integrated DNA Technologies) were used to target enrich for 139 or 425 predefined genes (Geneseeq Technology Inc.). The enriched libraries were sequenced on Hiseq 4000 NGS platforms (Illumina), while 168 and 520 lung cancer-relevant genes panel (Burning Rock Biotech) was performed using the Nextseq 500 (Illumina) platform. Libraries were quantified by qPCR using the KAPA Library Quantification kit (KAPA Biosystems). Library fragment size was determined by Bioanalyzer 2100 (Agilent Technologies). The mean coverage depth was 200X for the whole blood control samples, and 1000X for tumor tissues. For cfDNA samples, the mean coverage sequencing depth was 5000X.

Trimmomatic was used for FASTQ file quality control. Leading/trailing low quality (quality reading below 20) or N bases were removed. Paired-end reads were then aligned to the reference human genome (build hg19) using the Burrows-Wheeler Aligner (BWA) with the parameters. PCR deduplication was performed using Picard, the local realignment around indels and base quality score recalibration using GATK3. Matched tumor and normal sample pairs were first checked to have the same SNP fingerprint using VCF2LR (GeneTalk). Further, samples with mean deduplication depth <30X for blood and <600X for plasma were removed. Somatic Single Nucleotide Variant (SNV) calling was performed using Mutect, and insertion/deletions (INDELs) were called running Scalpel (scalpel-discovery in –somatic mode). SNVs and INDELs called were further filtered using the following criteria: i) minimum ≥5 variant supporting reads and ≥1% variant allele frequency (VAF) supporting the variant, ii) filtered if present in > 1% population frequency in the 1000g or ExAC database, iii) filtered through an internally collected list of recurrent sequencing errors (≥3 variant reads and ≤20% VAF in at least 30 out of ~2000 normal samples) on the same sequencing platform. The final list of mutations was annotated using vcf2maf (call VEP for annotation).

RNA-seq of 3 pre-transformation *EGFR/RB1*-mutant samples and 4 paired pre- and post-transformation *EGFR*-mutant/*RB1*-wide samples was conducted by Haplox Genomics Center. Firstly, total RNA from FFPE tissue was extracted using the RNeasy FFPE Kit (73504, Qiagen, Germany) following the manufacturer’s recommendations. The quality and purity of RNA were assessed by the NanoDroprmOne/Onec. Spectrophotometer (Thermo Scientific, MA, USA) and the Qubit RNA BRAssay Kit (Q10211, Invitrogen). Agilent4200 TapeStation system (Agilent, CA, USA) was used to identify RNA integrity. Subsequently, 1 µg of total RNA was prepared for library construction. RNA libraries were constructed using a VAHTSTM TotalRNA-seq (H/M/R) Library Prep Kit for Illumina (NR603, Wazyme). Then, Qubit 3.0 Fluorometer (Thermo Scientific, MA, USA) was used to measure the concentration of the constructed library, while Agilent 4200 TapeStation system was used to assess the fragment distribution. In addition, library molar concentration was measured on QuantStudio 5 qPCR system (Thermo Scientific, MA, USA). Finally, libraries were sequenced using NovaSeq 6000 S4 Reagent Kit V1.5 (20028312, illumina) on the illumina MovaSeaTM 6000 platform (San Diego, CA, USA). Differentially expressed genes were analyzed using edgeR. Differentially expressed genes were defined by an adjusted P value <0.05 and an absolute fold change >1. Gene set enrichment analysis (GSEA) was performed to calculate the normalized enrichment score^4^.

**Single-cell RNA dissociation**

Tissues were surgically removed and placed in MACS Tissue Storage Solution (Miltenyi Biotec) until processing. Following the outlined protocol, samples were washed with phosphate-buffered saline (PBS), finely minced (~1 mm^3^) on ice, and enzymatically digested using a mixture of 250 U/mL collagenase I (Gibco), 100 U/mL collagenase IV (Gibco), and 30 U/mL DNase I (Worthington) for 45 min at 37°C with agitation. Next, the digested tissue was then strained through a 70-µm cell strainer and centrifuged at 300 g for 5 minutes. After discarding the supernatant, and the pelleted cells were suspended in Red Blood Cell Lysis Buffer (Miltenyi Biotec), washed with PBS containing 0.04% BSA, and strained through a 35-μm cell strainer. Cell viability was assessed using Calcein-AM (Thermo Fisher Scientific) and Draq7 (BD Biosciences) staining ^5^.

**Single-cell RNA sequencing**

Single-cell RNA-seq and analysis were performed by NovelBio Bio-Pharm Technology Co., Ltd. The BD Rhapsody system was used to capture the transcriptomic information of the two sample-derived single cells. Single-cell capture was achieved by a random distribution of a single-cell suspension across > 200,000 microwells by a limiting dilution approach. Beads with oligonucleotide barcodes were added to saturation so that a bead was paired with each cell. The cells were lysed in the microwell to hybridize mRNA molecules to barcode capture oligos on the beads. Beads were collected in a single tube for reverse transcription and ExoI digestion. Each cDNA was tagged at the 5′-end (i.e., the 3′-end of an mRNA transcript) with a unique molecular identifier^6^ and cell barcode indicating its cell of origin. Whole-transcriptome libraries were prepared using the BD Rhapsody single-cell whole-transcriptome amplification workflow, including random priming and extension, RPE amplification PCR, and WTA index PCR. The libraries were quantified using a High-Sensitivity DNA Chip (Agilent) on a Bioanalyzer 2200 and the Qubit High-Sensitivity DNA assay (Thermo Fisher Scientific). Sequencing was performed using an Illumina sequencer (San Diego, CA, USA) on a 150-bp paired-end run. The details were fully described in a previous study^5^.

**Single-cell RNA analysis**

The scRNA-seq raw data were processed into FASTQ files^7^, mapped to the hg19 reference genome^8^, and the unique molecular identifier (UMI) counts were summarized into an expression matrix using CellRanger. Cell-level quality control was performed to filter cells by (1) total UMI counts no more than 1,000; (2) gene numbers no higher than 500; or (3) mitochondrial gene percentage over 20%. DoubletFinder (v.2.0.3) was applied to each sample, using principal components1-20, with nExp set to 0.08×nCells^2^/10,000, pN to 0.25, and pK to 0.09; cells identified as doublets were removed^9^. Subsequently, only cells with high-quality reads were retained for downstream analyses. For normalization and regression, we employed the Seurat package (version 3.1.4, https://satijalab.org/seurat/), using the functions of NormalizeData and ScaleData with the default parameters to mitigate the influence of sequencing library size, which converted expression values from UMI counts to ln[10,000 × UMI counts/total UMI counts in cell + 1]. After the standard Seurat clustering pipeline, cluster-level quality control was performed using the following functions in order: FindVariableFeatures with 2,000 genes, ScaleData, RunPCA, FindNeighbors with the first 20 PCs, and FindClusters with resolution 1, otherwise default settings. Clusters with average UMI counts of less than 1,500 were removed. We acquired the unsupervised cell cluster results and determined marker genes using the FindAllMarkers function with the Wilcox rank-sum algorithm and the following criteria: lnFC > 0.25, p < 0.05, min.pct > 0.1. Lastly, the Harmony package (version 1.0)^10^ of R was utilized to correct batch effects based on the top 50 PCA components identified.

**Copy number variation (CNV) estimation**

T cells were used as references to identify somatic CNVs using the R package CopyKAT with default parameters^11^. Cells exhibiting genome-wide CNVs were predicted as aneuploids, indicative of malignant cells, whereas those with minimal or low-magnitude CNVs were considered diploids, reflecting nonmalignant cells.

**Nomogram establishment, validation, and calibration**

The risk factors were examined by univariate and multivariate analyses (Binary logistic regression). Based on the results, a nomogram was formulated by R 4.0.5 (http://www.r-project.org) with the risk of SCLC transformation. A final model was selected using a backward step-down process, which used the Akaike information criterion as a stopping rule^12^. The model performance for predicting transformation was evaluated by calculating the concordance index (C-index) and ROC^13^. The value of the C-index ranges from 0.5 to 1.0, with 0.5 indicating a random chance and 1.0 indicating a perfect ability to discriminate the outcome with the model correctly. The discrimination of nomograms was verified using calibration plots. DCA is a method to evaluate the clinical utility of different predictive models^14^. It can compare the difference between nomogram and other models by quantifying the net income under different threshold probabilities. Since DCA can display the false- and the true-positive fractions as functions of the risk threshold, it compensates for any deficiency of ROC curves^15^.

**Statistical analysis**

Baseline characteristics were stratified according to different groups or T790M status and were compared by Pearson χ^2^-test. The ages of the patients between the groups were analyzed using independent samples T-test. Survival curves were obtained by Kaplan–Meier analysis. Risk and prognostic factors were examined by univariate and multivariate analyses (Binary logistic regression or Cox proportional hazards model). A P-value <0.05 was considered significant. All statistical analyses were carried out using SPSS 26.0 software (SPSS Inc., Chicago, IL, USA).

**References**

1. Travis WD, Brambilla E, Nicholson AG, et al. The 2015 World Health Organization Classification of Lung Tumors: Impact of Genetic, Clinical and Radiologic Advances Since the 2004 Classification. *J Thorac Oncol*. Sep 2015;10(9):1243-1260. doi:10.1097/JTO.0000000000000630

2. Raso MG, Bota-Rabassedas N, Wistuba, II. Pathology and Classification of SCLC. *Cancers (Basel)*. Feb 16 2021;13(4)doi:10.3390/cancers13040820

3. Marcoux N, Gettinger SN, O'Kane G, et al. EGFR-Mutant Adenocarcinomas That Transform to Small-Cell Lung Cancer and Other Neuroendocrine Carcinomas: Clinical Outcomes. *J Clin Oncol*. Feb 1 2019;37(4):278-285. doi:10.1200/JCO.18.01585

4. Offin M, Chan JM, Tenet M, et al. Concurrent RB1 and TP53 Alterations Define a Subset of EGFR-Mutant Lung Cancers at risk for Histologic Transformation and Inferior Clinical Outcomes. *J Thorac Oncol*. Oct 2019;14(10):1784-1793. doi:10.1016/j.jtho.2019.06.002

5. Yang L, He YT, Dong S, et al. Single-cell transcriptome analysis revealed a suppressive tumor immune microenvironment in EGFR mutant lung adenocarcinoma. *J Immunother Cancer*. Jan 2022;10(2)doi:10.1136/jitc-2021-003534

6. Paz-Ares L, Champiat S, Lai WV, et al. Tarlatamab, a First-in-Class DLL3-Targeted Bispecific T-Cell Engager, in Recurrent Small-Cell Lung Cancer: An Open-Label, Phase I Study. *J Clin Oncol*. Jun 1 2023;41(16):2893-2903. doi:10.1200/JCO.22.02823

7. Chen S, Zhou Y, Chen Y, Gu J. fastp: an ultra-fast all-in-one FASTQ preprocessor. *Bioinformatics*. Sep 1 2018;34(17):i884-i890. doi:10.1093/bioinformatics/bty560

8. Dobin A, Davis CA, Schlesinger F, et al. STAR: ultrafast universal RNA-seq aligner. *Bioinformatics*. Jan 1 2013;29(1):15-21. doi:10.1093/bioinformatics/bts635

9. McGinnis CS, Murrow LM, Gartner ZJ. DoubletFinder: Doublet Detection in Single-Cell RNA Sequencing Data Using Artificial Nearest Neighbors. *Cell Syst*. Apr 24 2019;8(4):329-337 e4. doi:10.1016/j.cels.2019.03.003

10. Korsunsky I, Millard N, Fan J, et al. Fast, sensitive and accurate integration of single-cell data with Harmony. *Nat Methods*. Dec 2019;16(12):1289-1296. doi:10.1038/s41592-019-0619-0

11. Gao R, Bai S, Henderson YC, et al. Delineating copy number and clonal substructure in human tumors from single-cell transcriptomes. *Nat Biotechnol*. May 2021;39(5):599-608. doi:10.1038/s41587-020-00795-2

12. Harrell FE, Jr., Lee KL, Mark DB. Multivariable prognostic models: issues in developing models, evaluating assumptions and adequacy, and measuring and reducing errors. *Stat Med*. Feb 28 1996;15(4):361-87. doi:10.1002/(SICI)1097-0258(19960229)15:4<361::AID-SIM168>3.0.CO;2-4

13. Harrell FE. *Regression modeling strategies: with applications to linear models, logistic regression, and survival analysis*. vol 608. Springer; 2001.

14. Chou WC, Rampanelli E, Li X, Ting JP. Impact of intracellular innate immune receptors on immunometabolism. *Cell Mol Immunol*. Mar 2022;19(3):337-351. doi:10.1038/s41423-021-00780-y

15. Balachandran VP, Gonen M, Smith JJ, DeMatteo RP. Nomograms in oncology: more than meets the eye. *Lancet Oncol*. Apr 2015;16(4):e173-80. doi:10.1016/S1470-2045(14)71116-7
